# Social incentivization of instrumental choice in mice requires amygdala-prelimbic cortex-nucleus accumbens connectivity

**DOI:** 10.1038/s41467-022-32388-9

**Published:** 2022-08-15

**Authors:** Henry W. Kietzman, Gracy Trinoskey-Rice, Sarah A. Blumenthal, Jidong D. Guo, Shannon L. Gourley

**Affiliations:** 1 Medical Scientist Training Program, Emory University School of Medicine, Atlanta, GA USA; 2 Department of Pediatrics, Emory University School of Medicine, Atlanta, GA USA; 3 Department of Psychiatry, Emory University School of Medicine, Atlanta, GA USA; 4 Graduate Program in Neuroscience, Emory University, Atlanta, GA USA; 5 Emory National Primate Research Center, Emory University, Atlanta, GA USA; 6 Children’s Healthcare of Atlanta, Atlanta, GA USA

**Keywords:** Social neuroscience, Motivation, Operant learning

## Abstract

Social experiences influence decision making, including decision making lacking explicit social content, yet mechanistic factors are unclear. We developed a new procedure, social incentivization of future choice (SIFC). Female mice are trained to nose poke for equally-preferred foods, then one food is paired with a novel conspecific, and the other with a novel object. Mice later respond more for the conspecific-associated food. Thus, prior social experience incentivizes later instrumental choice. SIFC is pervasive, occurring following multiple types of social experiences, and is not attributable to warmth or olfactory cues alone. SIFC requires the prelimbic prefrontal cortex (PL), but not the neighboring orbitofrontal cortex. Further, inputs from the basolateral amygdala to the PL and outputs to the nucleus accumbens are necessary for SIFC, but not memory for a conspecific. Basolateral amygdala→PL connections may signal the salience of social information, leading to the prioritization of coincident rewards via PL→nucleus accumbens outputs.

## Abstract (web summary)

Social experiences influence future decision making. The authors here establish a method for quantifying this phenomenon in mice and identify an amygdalo-frontal-striatal circuit controlling how social context shapes decisions.

## Introduction

Social information colors everyday choices (e.g., where to eat, what to wear) and major life decisions (e.g., which career to embark upon, with whom to partner). How mammals evolved to contend with the complexities of social information processing is intensively investigated^1–3^, and many mechanisms appear to be remarkably conserved from rodents to humans^4,5^. One challenge in fully understanding social information processing is that several parallel processes occur at once. For instance, to make a decision in a social context, a rodent must perceive (or remember) the sensory cues emitted by a conspecific, render those cues relevant, use those cues to motivate behavior, and initiate motor systems to generate a behavioral response. Elegant investigations of social recognition and approach have only reinforced the notion that social decision-making is complex and nuanced, likely necessitating novel strategies to untangle underlying neural circuits controlling particular processes. Here we attempt to understand how prior social experiences are integrated into future decisions. To do so, we developed a task, referred to as social incentivization of future choice (SIFC), whereby social experiences modulate later instrumental choice in mice—akin in humans to repeatedly returning to a particular restaurant because it’s where one had a first date.

A key node in the cognitive social brain of rodents is the medial prefrontal cortex (mPFC)^6^. The mPFC controls social approach^7^, representations of social partners^8^, and empathic-like processes^9^. In nonsocial contexts, the prelimbic subregion (PL) of the mPFC is necessary for rodents to learn associations between actions and their likely outcomes^10^, which is necessary for behavioral flexibility^11^ and inhibitory control^12^ when reward likelihood or contingencies change. The PL is thus uniquely positioned to control action selection, whether it is motivated by social experiences or nonsocial information.

Here, we find that the PL is necessary for social information to control instrumental choice, and we delineate key inputs and outputs. We contend that SIFC could be a useful tool in understanding how social experiences influence future action.

## Results

### Social information is used to guide future operant choice in mice

**Fig. 1:**
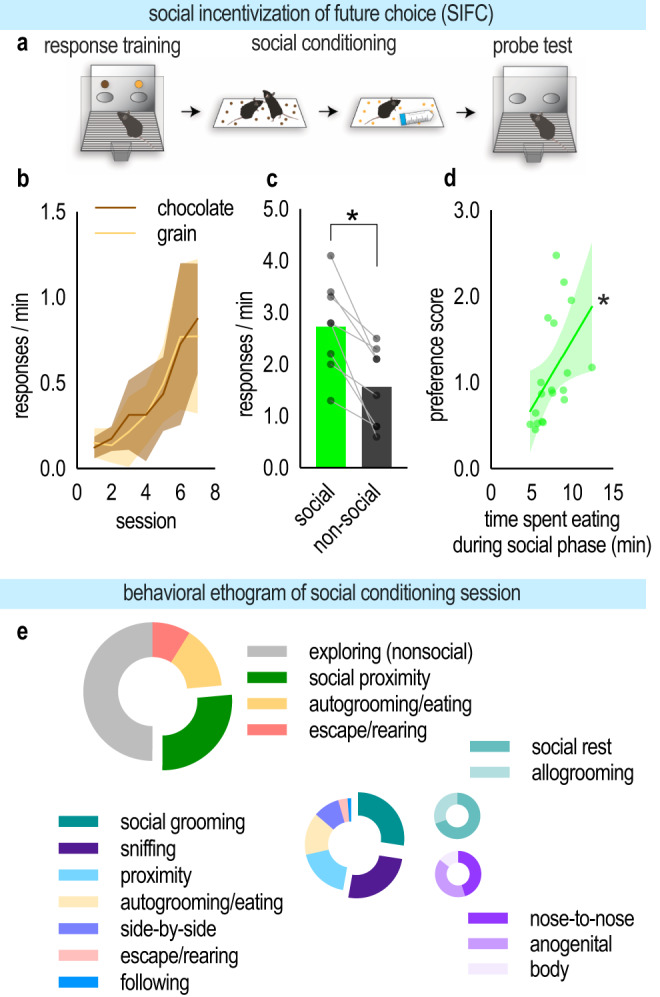
Social experience incentivizes future choice (SIFC). **a** Schematic of the SIFC task. Mice were trained to nose poke for two food reinforcers. Next, mice were placed in a chamber with either a novel conspecific or object and with one of the two reinforcers. The next day, each mouse received the other stimulus and pellet. Finally, mice were returned to the instrumental conditioning chambers used in training, and responding was quantified. **b** Response training; note no systemic preference for the chocolate or grain pellet. **c** Following the social conditioning phase, mice preferentially responded for the pellet associated with social experience [*n* = 8; paired *t*-test]. **d** The amount of time spent eating during the social conditioning phase predicted later preference for the pellet [calculated as response rates (social/nonsocial); *n* = 18 across multiple experiments; simple linear regression]. **e** (top left) Behavioral ethogram depicting behaviors observed during the social conditioning phase. For approximately ¼ of the social conditioning session, the experimental and stimulus mice were in social proximity, or within 1” of each other. (bottom right) Behavioral ethogram depicting behaviors exhibited when in social proximity. The two most commonly observed behaviors were variants of sniffing and grooming. Social rest refers to remaining immobile in close proximity to one another. Body refers to sniffing the body, as opposed to the anogenital region or engaging in nose-to-nose contact. Bars represent means, symbols represent individual mice. Line in (**d**) is simple linear regression. Shaded area in **b** = SEM, shaded area in **d** = 90% confidence interval. *p < 0.05. Source data are provided as a Source Data file.

Here, we aimed to understand how social information could inform future decision-making in rodent. To do so, we developed a task, SIFC, depicted in Fig. 1a. Female mice were trained to nose poke at two apertures for two distinct food reinforcers. Mice increased responding over time, acquiring the responses with no systemic preference for either pellet [main effect of the session (*F*_(6.84)_ = 20.89, *p* < 0.0001); no main effect of the pellet (*F* < 1)] (Fig. 1b). Response rates for both pellets are collapsed for simplicity in the rest of the report.

Next, mice were placed in a novel chamber with either a novel, same-age, female conspecific, or a novel object, and with one of the two reinforcers. Thus, one pellet was associated with a conspecific, while the other was associated with an object. When later returned to the instrumental conditioning chamber for a probe test, mice preferred the pellet associated with the social experience, which we will refer to as the social pellet, over the pellet associated with the falcon tube, referred to as the nonsocial pellet [paired *t*_(7)_ = 4.879, *p* = 0.0018] (Fig. 1c). Further, the amount of time spent eating during the social conditioning phase correlated with preference during the probe test [*r*^*2*^ = 0.2454, *p* = 0.0310] (Fig. 1d and Fig. S1). Other behaviors exhibited in the social conditioning phase are described in Table 1 and depicted in Fig. 1e. About a fourth of the time was spent in close proximity with the novel conspecific, for instance, sniffing and grooming. The frequency of eating was ~10x higher for experimental mice than stimulus mice, likely reflecting neophobia in the stimulus mice (for whom the pellet was novel).

**Table 1:** Description of behaviors analyzed in behavioral ethograms

**Nonsocial behaviors**	All behaviors were performed when the experimental mouse was not in social proximity to the stimulus mouse.
Explore	Exploration of the test chamber.
Autogroom/eating	Self-grooming and/or eating.
Escape/rearing	Rearing/ jumping behaviors along the edges of the test chamber.
**Social behaviors**	All behaviors were performed when the experimental mouse was in social proximity to the stimulus mouse.
Social rest	Experimental animal is groomed by the stimulus animal.
Allogrooming	Experimental animal grooms the stimulus animal.
Nose-to-nose sniffing	Experimental animal sniffs the stimulus animal’s nose.
Anogenital sniffing	Experimental animal sniffs the stimulus animal’s anogenital region.
Body sniffing	Experimental animal sniffs the observer animal’s body.
Autogroom/eat	Self-grooming and/ or eating.
Side-by-side	Experimental animals and stimulus animals sit beside each other.
Escape/rearing	Rearing/ jumping behaviors along the edges of the test chamber.
Following	Experimental animal follows closely behind the stimulus animal as it moves across the chamber.

Social proximity is defined as the time during which the experimental mouse is within a 1″ radius of the stimulus mouse. Autogrooming and eating were combined due to an inability to discern the two during recordings when the animal faced away from the camera.

### SIFC is pervasive and does not rely solely on olfactory or somatosensory cues

**Fig. 2:**
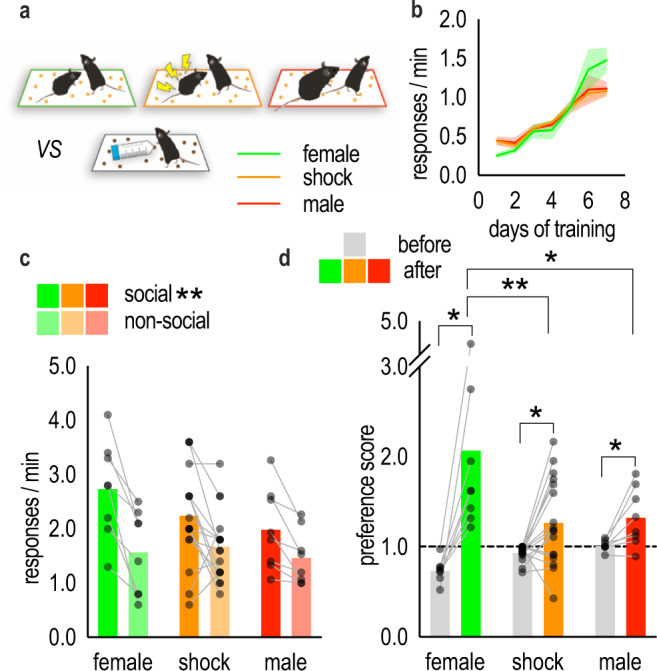
The ability of social experience to incentivize choice is pervasive. **a** Variants of the social conditioning phase of SIFC. Female mice were exposed to a same-age, same-sex novel conspecific (green, recapitulated from 1**c**), a same-age, same-sex novel shocked conspecific (orange), or a sexually-experienced unfamiliar male (red). **b** Response acquisition. **c** Following social conditioning, mice preferentially responded for the food associated with the social experience, regardless of the nature of that experience [*n*_female_ = 8, *n*_shock_ = 17, *n*_male_ = 8; two-way ANOVA]. **d** As expected, preference for that food increased relative to baseline, though a graded effect was apparent, such that preference was highest for the food associated with a same-age, same-sex conspecific [equivalent *n*’s to **c**; two-way ANOVA]. The dashed line depicts no preference. Acquisition curves represent means + SEMs. Bars represent means, symbols represent individual mice. *p < 0.05, **p < 0.001. Source data are provided as a Source Data file.

Thus far, we find that social experience with novel sex- and age-matched conspecific appears to confer value to an external reward, such that mice favor a reward associated with social experience. We next wanted to determine whether this phenomenon is pervasive—reflecting value conferred by the opportunity to investigate any conspecific, independent of the affiliative nature of the interaction—or restricted to particular interactions. To do so, we developed two variants of the social conditioning phase. In the shock variant, the novel conspecific was shocked immediately prior to social conditioning using a procedure that triggers stress hormone release in the observer mouse^13^. In the male variant, the novel conspecific was a sexually experienced, retired breeder male (Fig. 2a).

Mice learned to nose poke for food reinforcers [*F*_(6,198)_ = 54.03, *p* < 0.001] (Fig. 2b), then one reinforcer was paired with one of the three conspecifics. During the later probe test, mice preferred the pellet associated with the novel conspecific. A main effect of nose poke aperture was detected, indicating that multiple types of social experiences incentivize later responding [main effect of nose poke aperture (*F*_(1,30)_ = 32.30, *p* < 0.0001), no main effect of group (*F* < 1), no group*behavior interaction (*F*_(2,30)_ = 2.270, *p* = 0.1208)] (Fig. 2c).

The same data can be converted into response preferences (responses for the social/nonsocial pellet). Scores prior to social conditioning were ∼1, reflecting no preference, and preference developed following social conditioning as expected; however, a graded effect of conspecific type was apparent, in that preference was greatest following an experience with an unshocked, same-sex conspecific [group*time interaction (*F*_(2,30)_ = 7.248, *p* = 0.0027), main effect of time (*F*_(1,30)_ = 30.23, *p* < 0.0001), no main effect of group (*F*_(2,30)_ = 1.950, *p* = 0.1599)] (Fig. 2d). These and all other post hoc comparisons are indicated in the figures.

**Fig. 3:**
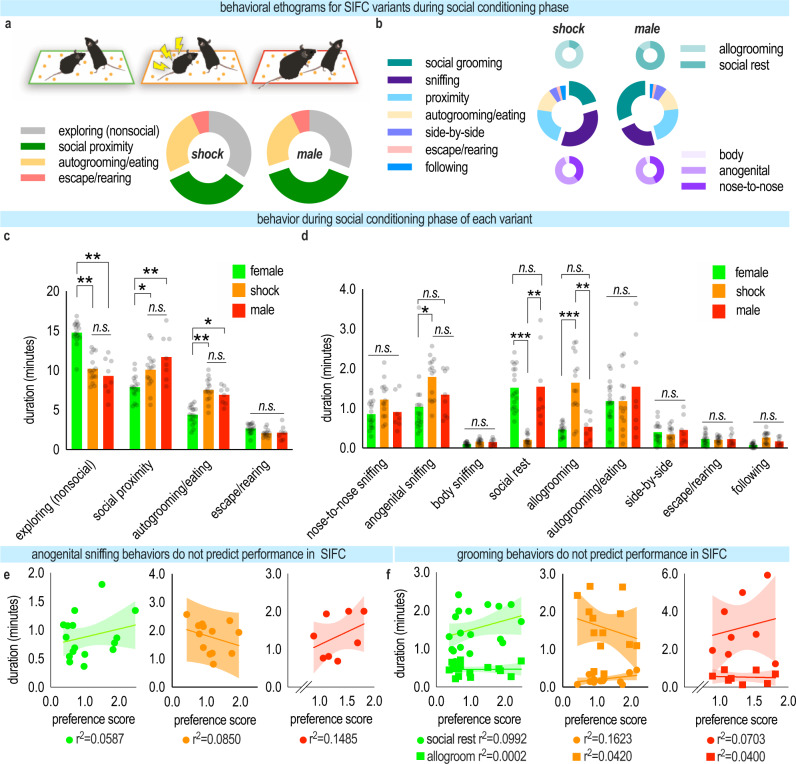
Characterization of behavior during social conditioning. **a** (top) Experimental mice interacted with naïve female, shocked female, or male conspecifics. (bottom) Ethogram depicting behaviors exhibited by the experimental mouse when paired with a shocked (left) and male (right) conspecific. The naïve female condition is depicted in Fig. 1. **b** Ethogram depicting all behaviors exhibited by the experimental mouse while in social proximity of the shocked (left) or male (right) conspecific. Social proximity was defined as a distance of 1″ between animals. **c** Behaviors exhibited by the experimental mouse during each SIFC stimulus variant during the social conditioning phase. The experimental mouse in the shock or male condition spent more time in social proximity and more time eating than those in the female condition [*n*_female_ = 18, *n*_shock_ = 15, *n*_male_ = 8; two-way mixed-model ANOVA] **d** Behaviors exhibited by the experimental mouse during each SIFC variant while in social proximity during the conditioning phase. In social proximity, significant differences were observed in sniffing and grooming behaviors [equivalent *n*’s to **c**; two-way mixed-model ANOVA]. **e** Anogenital sniffing does not predict response preference in SIFC [simple linear regression] **f** Grooming behaviors also do not predict response preference in SIFC [simple linear regression]. Bars represent means + SEMs, and symbols represent individual mice, lines represent simple linear regressions, shaded areas represent 90% confidence intervals. *p < 0.05, **p < 0.001. Source data are provided as a Source Data file.

What factors might account for SIFC? We observed and quantified several behaviors in the social conditioning phase. Mice encountering a shocked or male conspecific behaved generally similarly (Fig. 3a, b). Quantification of various behaviors across all three conditions (i.e., same-sex non-shocked conspecific, shocked conspecific, retired breeder male) did, however, reveal some differences that might account for a graded effect in SIFC, depending on the variant. In particular, mice paired with a same-sex non-shocked conspecific spent more time investigating the testing chamber than the other groups [group*behavior interaction (*F*_(6,149)_ = 28.73, *p* < 0.0001), main effect of behavior (*F*_(3,149)_ = 246.6, *p* < 0.0001), no main effect of group (*F* < 1)] (Fig. 3c). Meanwhile, other behaviors varied non-systematically [group*behavior interaction (*F*_(16,284)_ = 10.65, *p* < 0.0001), main effect of behavior (*F*_(8,284)_ = 45.55, *p* < 0.0001), no main effect of group (*F*_(2,38)_ = 2.260, *p* = 0.1182)] (Fig. 3d). Conceivably, this exploration time allowed the mice to form strong associations between the pellets scattered throughout the chamber and the social experience—translating to strong SIFC.

A reasonable expectation is that social behaviors like anogenital investigation, allogrooming, and social rest (meaning, spending time immobile in close proximity to the conspecific) might predict SIFC, but we did not find evidence of this. Specifically, mice encountering a same-sex non-shocked conspecific—those with the greatest SIFC—did not engage in these behaviors any more than other groups (as one might predict), and none of these behaviors predicted SIFC in any of the groups [*p* > 0.05] (Fig. 3e, f).

**Fig. 4:**
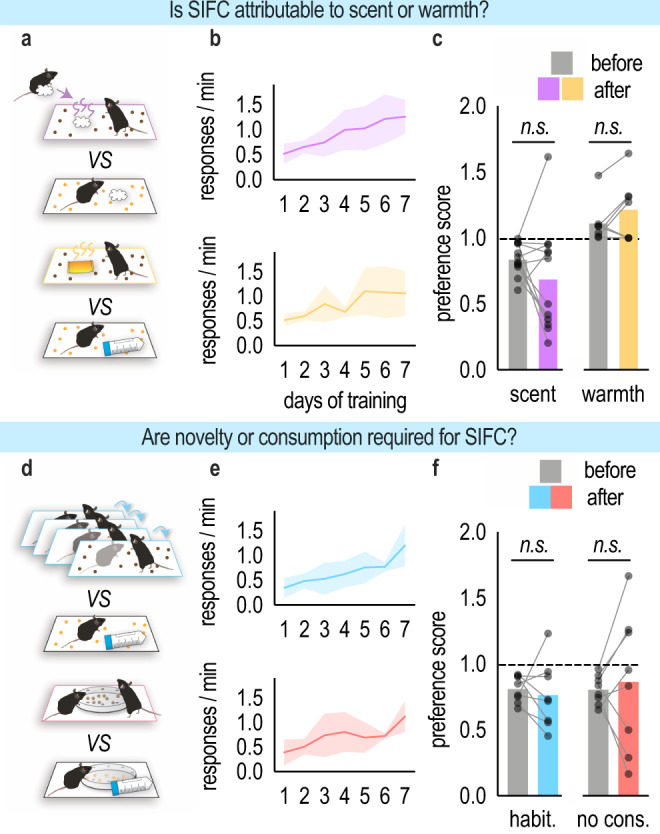
SIFC does not rely on sensory properties like scent or warmth and requires a novel social experience combined with consumption. **a** (above) To determine whether SIFC is attributable to the scent of a conspecific, one of the pellets was paired with a cotton swab that had been rubbed on a novel conspecific, while the other was paired with a fresh cotton swab. (below) To determine whether SIFC is attributable to the warmth derived from huddling, one of the pellets was paired with a heating pad, and the other was paired with a falcon tube. **b** Response acquisition. **c** Neither scent nor warmth modified later instrumental response preferences [*n*_scent_ = 12, *n*_warmth_ = 7; paired *t*-test]. **d** (above) To determine whether SIFC requires a novel social interaction, one of the pellets was paired with a conspecific to which the experimental mouse had been habituated, and the other was paired with a novel object. (below) To determine whether SIFC requires mice to eat during the social experience, pellets were present but inaccessible during the social conditioning phase. **e** Response acquisition. **f** Exposure to a familiar conspecific and obstruction of pellet consumption failed to generate SIFC [*n*_habit._ = 8, *n*_no cons._ = 8; paired *t*-test]. A dashed line indicates no preference. Acquisition curves represent means + SEMs. Bars represent means, symbols represent individual mice. Habit. habituation, no cons. no consumption. Source data are provided as a Source Data file.

Our findings suggest that social experiences influence later choice, though they do not preclude the possibility that some sensory quality of the social experience, such as scent or warmth, accounts for SIFC. To test this possibility, we next trained mice to respond to food pellets, then paired one of the pellets with a cotton swab that had been rubbed on a novel, same-age, same-sex conspecific using a procedure that can cause avoidance or approach in other social conditioning tasks^9^. The other pellet was paired with a clean cotton swab (Fig. 4a). In a second variant, one pellet was paired with a heating pad in an effort to recapitulate warmth from huddling with the stimulus mouse, while the other pellet was paired with a novel object (Fig. 4a). In both experiments, mice acquired the nose poke response [to be scent condition: main effect of the day (*F*_(6,66)_ = 10.60, *p* < 0.0001); to be warmth condition: main effect of the day (*F*_(6,36)_ = 4.714, *p* = 0.0011)] (Fig. 4b), but they did not later develop a pellet preference [scent (*t*_(11)_ = 1.342, *p* = 0.2068); warmth (*t*_(6)_ = 1.688, *p* = 0.1463)] (Fig. 4c). Thus, olfactory cues or warmth alone do not appear to account for SIFC.

Next, we wanted to better understand which elements of the social experience incentivize later choice. First, we developed another variant of SIFC during which the social experience was no longer novel by habituating the experimental mouse to the stimulus mouse over the course of three days (Fig. 4d). We also devised a variant in which we made pellets inaccessible during the social experience, thereby dissociating the act of consuming the pellet from investigating the conspecific (Fig. 4d). In both experiments, mice acquired the nose poke response [to be habituation condition: main effect of the day (*F*_(6,42)_ = 13.55, *p* < 0.0001); to be no consumption condition: main effect of the day (*F*_(6,42)_ = 6.583, *p* < 0.0001)] (Fig. 4e). Following social conditioning, mice displayed no systematic preference for the pellet associated with the conspecific [habituation (*t*_(7)_ = 0.0597, *p* = 0.96); consummatory control (*t*_(7)_ = 0.4565, *p* = 0.662)] (Fig. 4f). Thus, novelty and the act of consumption (rather than being able to smell the food, for example) are required for SIFC.

### The PL and its connections with the basolateral amygdala (BLA) and nucleus accumbens (NAc) are necessary for SIFC

**Fig. 5:**
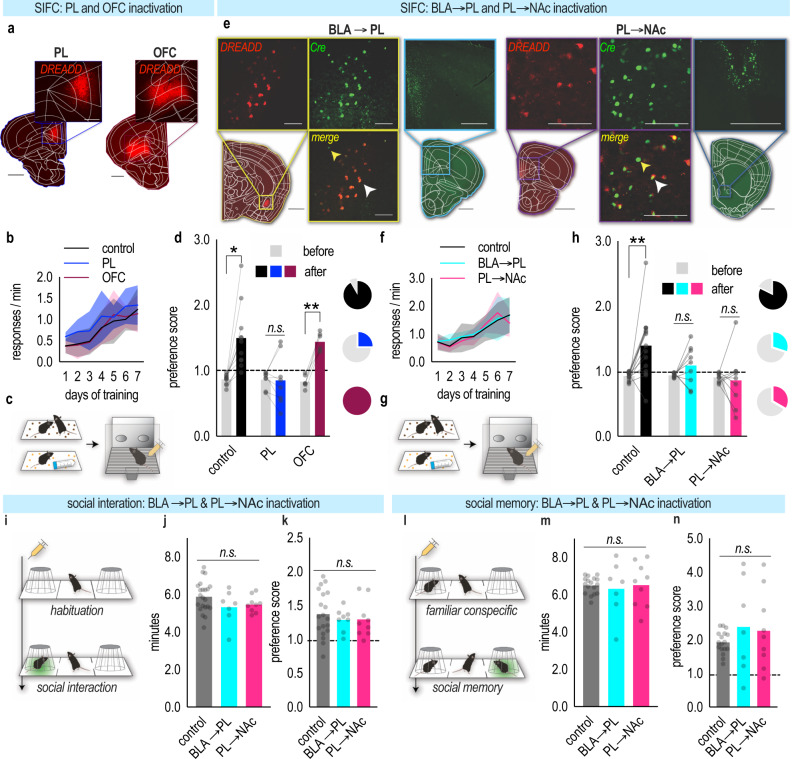
BLA→PL and PL→NAc connections control SIFC, but not sociality nor social memory. **a** Viral vectors expressing Gi-DREADDs or mCherry in the PL or OFC. Scale bar = 1 mm, 0.5 mm (inset) **b** Response acquisition. Control groups did not differ and were combined. **c** Task conducted as in Fig. 1, except CNO, was administered prior to the probe test (syringe). **d** Following social conditioning, control and OFC mice responded preferentially for the food associated with the social experience. PL inactivation abolished this preference. Pie charts represent the percentage of mice/group that preferred the food previously paired with a conspecific [*n*_control_ = 11, *n*_PL_ = 8, *n*_OFC_ = 6; two-way ANOVA] **e** Gi-DREADDs were expressed in BLA→PL or PL→NAc projections. (left) Representative histology. Red in the BLA depicts the Cre-dependent Gi-DREADDs-mCherry; green depicts retrograde-Cre-GFP in the PL. Scale bar = 1 mm, 0.5 mm (inset) (right) Representative histology. Red in the PL depicts the Cre-dependent Gi-DREADDs-mCherry; green depicts retrograde-Cre-GFP in the NAc. The white arrowhead indicates co-localization of Cre-dependent mCherry-Gi-DREADDs with Cre-GFP; the yellow arrowhead indicates a Cre+ neuron with no mCherry-Gi-DREADDs expression. Of note, mCherry is not present in the absence of Cre. Scale bar = 1 mm, 100 µm (inset) **f** Response acquisition. **g** Again, CNO was administered prior to the probe test. **h** Control mice responded for food associated with a social experience. Projection inactivation ablated preference. Pie charts represent the percentage of mice/group that demonstrated preferential responding [*n*_control_ = 15, *n*_BLA→PL_ = 9, *n*_PL→Nac_ = 9; two-way ANOVA]. **i** Three-chamber interaction task: Following habituation, two chambers are revealed, containing a novel conspecific or empty cup. **j** Silencing BLA→PL or PL→Nac projections did not affect investigation time with the novel conspecific [*n*_control_ = 22, *n*_BLA→PL_ = 8, *n*_PL→Nac_ = 9; Welch’s ANOVA], nor **k** preference for the conspecific [*n*_control_ = 19, *n*_BLA→PL_ = 8, *n*_PL→Nac_ = 9; one-way ANOVA]. **l** Social memory task schematic: A novel conspecific is placed under the previously empty cup. **m** Projection silencing had no effect on investigation time [*n*_control_ = 17, *n*_BLA→PL_ = 7, *n*_PL→Nac_ = 9; Welch’s ANOVA] nor **n** preference [equivalent *n*’s to **m**; Welch’s ANOVA]. Throughout, the dashed line depicts no preference. Acquisition curves represent means + SEMs. Bars represent the means and symbols individual mice. *p < 0.05, **p < 0.001. Source data are provided as a Source Data file.

We hypothesized that the PL controls the ability of social experience to incentivize future action selection. To test this possibility, we infused either a control viral vector expressing mCherry or a viral vector expressing Gi-coupled Designer Receptors Exclusively Activated by Designer Drugs (DREADDs) into the PL or the neighboring orbitofrontal cortex (OFC) for comparison (Fig. 5a and Fig. S2). Mice acquired food-reinforced nose poke responses [main effect of the day (*F*_(6,132)_ = 32.18, *p* < 0.0001) no main effect of group (*F*_(2,22)_ = 1.581, *p* = 0.2283), no day*group interaction (*F* < 1)] (Fig. 5b), and one pellet was paired with a novel conspecific as above. SIFC was then conducted as before in Fig. 1, only this time all mice, regardless of group, were administered the Gi-DREADDs ligand clozapine *N*-oxide (CNO) prior to a probe test (Fig. 5c). Inactivating the PL, but not OFC, ablated SIFC [group*aperture interaction (*F*_(2,22)_ = 7.962, *p* = 0.0025), main effect of aperture (*F*_(1,22)_ = 28.38, *p* < 0.0001), main effect of group (*F*_(2,22)_ = 5.272, *p* = 0.0135)] (Fig. 5d).

We next sought to determine the PL connections necessary for SIFC. To manipulate BLA→PL projections, we infused a viral vector expressing a retrograde-Cre recombinase (Cre) + green fluorescent protein (GFP) into the PL and a viral vector expressing a Cre-dependent Gi-DREADDs in the BLA (Fig. 5e and Fig. S2). As such, the Cre will travel retrogradely to the BLA and permit the Gi-DREADDs to be transcribed in BLA→PL projections. To confirm the effectiveness of projection-specific Gi-DREADDs, whole cell patch clamp recordings were obtained from BLA neurons (Fig. S3). In Gi-DREADDs-mCherry(+), but not Gi-DREADDs-mCherry(−), neurons, bath application of CNO induced rapid and sustaining membrane hyperpolarization, increased rheobase (the minimum current required to generate a single action potential), and action potential firing, as expected (Fig. S3).

To similarly manipulate PL→NAc projections, we infused a retrograde-Cre+GFP into the NAc and Cre-dependent Gi-DREADDs in the PL (Fig. 5e and Fig. S2). Mice acquired the food-reinforced nose poke responses [main effect of day (*F*_(6,180)_ = 32.10, *p* < 0.0001), no other main effects or interactions (*F*s < 1)] (Fig. 5f). SIFC was then conducted as before, with the administration of CNO (yellow syringe) prior to a probe test (Fig. 5g). Following social conditioning, control mice preferred the social pellet, while inactivation of BLA afferents or NAc efferents of the PL ablated this preference [group*aperture interaction (*F*_(2,30)_ = 3.941, *p* = 0.0302), main effect of aperture (*F*_(1,30)_ = 5.658, *p* = 0.0239), main effect of group (*F*_(2,30)_ = 5.099, *p* = 0.0124)] (Fig. 5h). Thus, SIFC requires BLA→PL and PL→NAc connections. The posterior PL may be particularly critical, given that investigation of a social conspecific relative to a novel item triggers more immediate-early gene expression in this subregion (Fig. S4).

BLA→PL and PL→NAc connections could conceivably be conveying: (1) information key to sociality, referring to the ability to prioritize or attend to social stimuli, or (2) social memory, that is, memory for a social experience. Disruption of either of these processes could lead to deficits in SIFC. To explore these possibilities, we turned to a three-chamber social interaction test (Fig. 5i). In the first phase of this task, mice can investigate a novel conspecific or an empty cup; typical mice will prefer the novel conspecific, reflecting intact sociality. Inactivation of BLA→PL and PL→NAc projections did not affect time with the novel conspecific [Welch’s ANOVA: *W*_(2,20.54)_ = 1.696, *p* = 0.2081] (Fig. 5j), nor preference for the conspecific over the empty cup [one-way ANOVA: *F* < 1] (Fig. 5k).

In the second phase of this task, a new, unfamiliar conspecific replaces the cup; typical mice will spend more time investigating an unfamiliar conspecific, reflecting intact social memory for the familiar conspecific (Fig. 5l). Inactivating BLA→PL and PL→NAc projections did not affect the amount of time spent with an unfamiliar conspecific [Welch’s ANOVA *W*_(2,10.07)_ = 0.0513, *p* = 0.9502] (Fig. 5m). Further, preference for the unfamiliar conspecific was also unchanged [Welch’s ANOVA *W*_(2,9.725)_ = 0.6910, *p* = 0.5241] (Fig. 5n). Thus, BLA afferents and NAc efferents of the PL appear to convey the salience of social memory in SIFC, but are not involved in the social approach, or in remembering that social interaction, per se.

### Immediate-early gene content in the BLA associated with individual differences in SIFC

**Fig. 6:**
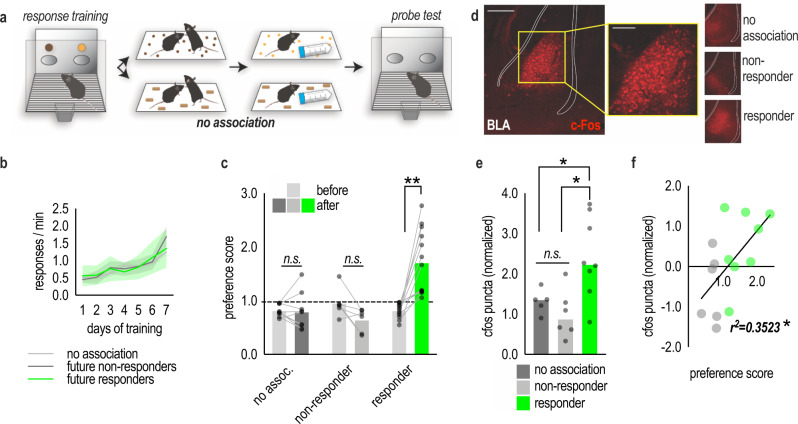
C-Fos in the BLA predicts individual differences in SIFC. **a** Schematic of the task. Mice were trained to nose poke for two food reinforcers. Mice were then conditioned as before (top), or, in the no association group (bottom), normal chow replaced the reinforcer pellets. Mice were returned to the operant conditioning chambers used in training, and responding to the reinforcers was quantified. **b** To capitalize on individual differences in SIFC, we retroactively subdivided the mice based on their performance during the probe test: those that exhibited social preference were deemed responders, and those that did not were deemed nonresponders. Mice acquired the nose poke responses. **c** Following the social conditioning phase, responders preferentially responded to the pellet associated with the social experience, while nonresponders and mice in the no association cohort exhibited no preference, as expected [across multiple experiments *n*_no assoc._ = 17, *n*_non-resp._ = 7, *n*_resp._ = 13; two-way ANOVA]. **d** Representative images depicting c-Fos in the BLA following SIFC. Scale bar = 200 µm. **e** C-Fos levels were higher in responders than nonresponders or the no association group [*n*_no assoc._ = 5, *n*_non-resp._ = 6, *n*_resp._ = 8; one-way ANOVA]. **f** The amount of c-Fos in the BLA predicted the amount of preference for the social pellet in SIFC [*n* = 14]. A dashed line indicates no preference. Bars represent means, a line represents simple linear regression, symbols represent individual mice, in (**b**) shaded area represents SEMs. *p < 0.05, **p < 0.001. No assoc. no association, non-resp. non-responder, resp. responder. Source data are provided as a Source Data file.

In a final experiment aimed at understanding individual differences in SIFC, mice were trained to nose poke at two apertures for two distinct food reinforcers, as above. We retroactively subdivided the mice based on their performance during the probe test: those that exhibited social preference were deemed responders; those that did not were deemed nonresponders. In a final group, deemed the no association group, the pellets in the social conditioning phase were replaced with familiar chow (Fig. 6a). As such, these mice still experienced handling, instrumental conditioning, interaction with a novel conspecific, etc., but not the opportunity to associate these experiences with the food reinforcers used for instrumental conditioning. There were no group differences in initial training [main effect of day (*F*_(6,162)_ = 21.72, *p* < 0.0001); no main effect of group or no day*group interaction effect (*F*s < 1)] (Fig. 6b). At the probe test following social conditioning, responders displayed a preference for the social pellet, as expected, while nonresponders and mice in the no association group had no preference [group*nose poke aperture interaction (*F*_(2,25)_ = 15.93, *p* < 0.0001); main effect of group (*F*_(2,25)_ = 11.889, *p* = 0.0002); no main effect of aperture (*F*_(1,25)_ = 3.608, *p* = 0.069)] (Fig. 6c). Interestingly, the future nonresponders consumed more food during the social conditioning phase than the future responders, strongly suggesting that poor feeding cannot account for insensitivity (Fig. S5).

C-Fos is a neuronal immediate-early gene induced by membrane depolarization and cell firing^14^. As such, it is considered a marker of neuronal activity. We quantified c-Fos in the BLA, revealing high levels in responders; meanwhile, nonresponders were indistinguishable from mice that never formed associations [*F*_(2,16)_ = 6.724, *p* = 0.0076] (Fig. 6d, e). Further, c-Fos levels predicted SIFC, such that higher levels correlated with the greatest preference for the pellet associated with the novel conspecific [*r*^2^ = 0.3523, *p* = 0.0252] (Fig. 6f). These findings corroborating our chemogenetic experiments indicating that the BLA is a key node by which associations between social interactions and external rewards form.

## Discussion

Across species, social influences guide choices. Here, we established a task, SIFC, whereby mice use social information to guide a future choice: Mice are trained to respond in instrumental conditioning chambers for two food pellets, then one pellet is paired with social interaction. We find that mice will later respond preferentially to that pellet, providing us with an opportunity to understand how social experiences incentivize future choices. We find that SIFC requires the PL and its BLA afferents and NAc efferents; these projections are not necessary for sociality or social memory. Moreover, immediate-early gene levels in the BLA predict individual differences in SIFC, suggesting that the BLA primes organisms to seek rewards associated with prior social experiences.

### Social information guides future choice

The ability of rodents to perceive or recognize social conspecifics has been the subject of investigation for decades (reviewed in ref. 15), but how rodents use social information to guide future choices is less clear, and understanding this process likely requires new tools and tasks. Our task, SIFC, tests the ability of a social experience to confer value to an external reinforcer, influencing future choices. We find that SIFC occurs following experience with a shocked conspecific, which elicits a stress response in the observer^13^, a female conspecific, or a sexually-experienced unfamiliar male. Our interpretation is that the opportunity to gain novel social information, writ large, is adaptively valuable and that SIFC reflects that value rather than strictly the affiliative properties of the social interaction itself. This notion is reinforced by the finding that SIFC does not occur with familiar conspecifics. Adaptive value is then transferred to external reward, which causes the mouse to seek that reward in the future. In this report, SIFC was conducted solely in females to limit conspecific-directed aggression in males. Future work could investigate aggressive social interactions, though, which are highly information-rich and can even be reinforcing^16^.

Importantly, SIFC differs from tasks examining the social transmission of food preferences (STFP) in that it measures responding to a non-novel food following its association with a social experience. Meanwhile, STFP involves the transmission of a safety signal from a conspecific, which has previously encountered the food to an experimental subject, which has not and would typically suppress their consumption due to innate neophobia^17^. In SIFC, the conspecific has never encountered the pellet and thus cannot transmit any information regarding its safety to the experimental subject. Moreover, the experimental subject is already highly experienced with the pellet due to instrumental response training.

### SIFC requires BLA→PL and PL→NAc connections

The rodent PL is considered a central hub of the rodent so-called social brain;^6,18^ for example, investigation of a social target leads to increased neuronal firing in the PL^7^, and disrupting the excitatory/inhibitory balance of the PL impairs social interaction^19^. It is perhaps unsurprising, then, that inactivation of the PL eliminated SIFC, such that mice appeared as if they had had no prior social experience.

We next revealed that the inactivation of BLA→PL connections obstructed SIFC. Neurophysiological studies suggest that the BLA contains representations of both positive and negative changes in outcome value^20^, and rapid glutamate release in the BLA is required for reward-predictive cues to motivate goal-directed action^21^. Accordingly, the BLA is necessary for Pavlovian-to-instrumental transfer (PIT)^22–24^, referring to the capacity of cues associated with a reward to motivate instrumental responding for that same reward^23^. SIFC bears similarity to PIT in that the social experience is linked with a food pellet via Pavlovian associations, and instrumental responding to that food is later energized. We envision that the BLA serves similar functions in PIT and SIFC, incentivizing operant responding as mice recall memories linking social interaction with the pellet. This notion led us to predict that immediate-early gene expression in the BLA should predict individual differences in SIFC, which was indeed the case, such that mice with robust SIFC displayed the most robust c-Fos in the BLA.

One difference between SIFC and PIT is that in PIT, cue presentation occurs during the probe test and energizes in-the-moment responding relative to test epochs when the cue is not present. By contrast, in SIFC, the conspecific is not present in the probe test, so responding is energized, unprompted, by memory for previously established associations. This difference may account for the evidence that classical PIT requires the OFC but not PL^10,25,26^, while we discovered the inverse – that is, that SIFC requires the PL and not OFC. This difference may reflect the capacity of SIFC to trigger value memory processing, a function closely tied to the PL^27^, rather than in-the-moment response plasticity, a function often ascribed to the OFC.

We next asked which PL outputs control SIFC. PL→NAc projections are required for a motivated approach, referring to seeking behavior that follows reward-predictive cues^28^. Individual cortico-striatal neurons tune their responses to reward-predictive cues during Pavlovian conditioning, and stimulation of PL→NAc neurons increases conditioned reward-seeking behavior^29^. In social contexts, the reinforcing properties of social interaction, assayed by socially conditioned place preference, require oxytocinergic activity in the NAc^30^. Finally, PL→NAc projections encode socio-spatial associations in a modified three-chamber task, meaning that a large proportion of PL→NAc neuronal ensembles preferentially fire in response to a social conspecific in one spatial location (either the right or left side of the chamber)^31^. We found that PL→NAc projections are required for mice to seek a food reward that had been associated with a conspecific, presumably incentivizing seeking behavior based on historical social experience.

Together, our findings provide clear, converging evidence that excitatory neurons in the PL control action selection based on prior social interactions. How the PL impacts social interaction itself remains unclear. We found that inactivating PL connectivity with the BLA and NAc did not impact social interaction in a three-chamber interaction task. Other studies also observed no effects^32^, or even increased social investigation^33^, with general PL inactivation strategies. The mPFC, and the PL, in particular, is a remarkably heterogeneous structure. Recent work by Lin and colleagues demonstrated that the PL contains distinct ON and OFF excitatory neural ensembles when subjects freely explored restrained social targets. While ON neurons increased their firing in response to a social target, OFF neurons suppressed their firing^34^. Dynamic ON and OFF populations are likely both inactivated in global inhibition studies, obscuring the specific functions of distinct ensembles.

The PL occupies a large territory, with topographically organized inputs and outputs, leading us to quantify c-Fos+ puncta in the anterior *vs*. posterior compartments. Social interaction triggered more c-Fos in the posterior compartment than experience with a novel nonsocial stimulus (in this case, a novel food), while c-Fos labeling was comparable in the anterior compartment. Relative to the anterior PL, the posterior PL receives projections from the ventral pallidum and claustrum^35^, and projects to the anterior cingulate cortex (ACC)^36^. The ACC is involved in multiple social processes in rodents, including social recognition^37^, the social transmission of fear^38^ and pain^39^, and even consolation-like behavior^40^. Whether ACC-posterior PL connections are involved in SIFC (and potentially, not decisions lacking social content) could be empirically tested in future investigations. Another consideration in our study is that PL-to-striatum neurons arborize in a highly stereotyped pattern, with collaterals terminating in the brainstem, spinal cord, claustrum, and striatum itself;^41^ these collaterals could be functionally examined in future investigations. Relatedly, BLA→PL projections can collateralize to the nucleus accumbens^42^, but recent investigations approximate that only ~5% of BLA efferents project to multiple areas^43^. This pattern suggests that the behavioral consequences of BLA→PL projection inactivation here were unlikely to be attributable to off-target effects.

In all, our findings indicate that (1) prior social experiences can inform decision-making in rodents, (2) utilizing BLA→PL and PL→NAc connections. Deficits in social cognition emerge in almost every neuropsychiatric disease^44^. SIFC may be useful for better understanding how social experiences incentivize future behaviors and how this process goes awry in neuropsychiatric illness.

## Methods

### Subjects

Procedures were approved by the Emory University IACUC. Experimental mice were female C57BL/6 mice 2–6 months of age. We used females to minimize conspecific-directed aggression. Stimulus mice (that is, mice used as novel conspecifics) were unfamiliar with same-strain females within 1 month of age. The one exception is an experiment in which we used mature, unfamiliar breeding males (aged 6–12 months) from our colony, also bred on a C57BL/6 background. Otherwise, mice were randomized into groups. All mice were bred from Jackson Labs stock, maintained on a 12 h light cycle (0700 on), with temperature and humidity ranges of 17.8–26.1 °C and 30–70%, respectively. Subjects were provided food and water ad libitum unless otherwise noted. We did not test the estrous cycle for this study.

### Stereotaxic surgery and viral vectors

Mice were anesthetized with ketamine/dexmedetomidine (100/0.5 mg/kg, intraperitoneal injection (i.p.), 1 ml/100 g) and placed in a digitized stereotaxic frame (Stoelting). Small holes were drilled in the skull, and viral vectors were infused at AP + 1.7, ML ± 0.17, DV-2.5 (PL); AP + 2.6, ML ± 1.2, DV-2.8 (OFC); AP-1.4, ML ± 3.0, DV-4.9 (BLA); or AP + 2.5, ML ± 0.95, DV-4.20 (NAc). Viral vectors were infused over five min in a volume of 0.5 μl. Viral vector details are described in Table 2. Syringes were left in place for ≥5 min for PL infusions, or ≥8 min for BLA/NAc infusions, prior to removal and suturing. Mice were revived with antisedan (3 mg/kg, i.p.) and left undisturbed for at least 3 weeks prior to behavioral experiments.

**Table 2:** Viral vectors used in each experiment

Experiment	Viral vectors	Supplier
PL or OFC regional inactivation	AAV5-CaMKII-hM4Di-Gi-mCherry or AAV5-CaMKII-mCherry	UNC Viral Vector Core
BLA→PL projection inactivation	rgAAV(pENN)-hSyn-HI-eGFP-Cre-WPRE (PL) AAV5-hSyn-DIO-mCherry±hM4D(Gi) (BLA)	Addgene
PL→NAc projection inactivation	rgAAV(pENN)-hSyn-HI-eGFP-Cre-WPRE (NAc) AAV5-hSyn-DIO-mCherry±hM4D(Gi) (PL)	Addgene

### Instrumental response training

First, mice were singly housed. The next day, mice were food restricted to ~90% of their free-feeding body weight to motivate food-reinforced responding. Mice were then trained to nose poke for two distinct food reinforcers that are equivalently preferred (see again the first figure) (20 mg Bio-Serv Dustless Precision Pellets, grain and chocolate) in Med Associates operant conditioning chambers equipped with two nose poke apertures and a separate food magazine. Responding on each aperture was reinforced using a fixed ratio 1 (FR1) schedule of reinforcement, such that 30 pellets were available for responding on each aperture. Sessions ended at 70 min, or 60 pellets acquired. Training sessions were conducted once daily and ended at seven sessions, or when mice acquired all 60 pellets within 70 min (if mice required greater than seven sessions). Response acquisition curves represent responses/min during the last seven training sessions, with no side preferences throughout.

### Social incentivization of future choice (SIFC)

Mice were trained to nose poke for food as above. Then, social conditioning occurred. First, mice were habituated to testing chambers (large, clean, empty cages) by allowing them to explore under low light with no stimuli present for 30–60 min. The social conditioning phase of the task consisted of two sessions (1/day), which were counterbalanced in order. In one session, the experimental mouse was placed in the chamber with a novel conspecific and either the grain or chocolate pellet. In the other session, the mouse was placed in the same chamber with the other pellet and a novel object, a 15 mL falcon tube. Pellets were weighed before and after each session to ensure that a minimum of 0.2 g of pellets were consumed. If 0.2 g were not consumed, the pairing session was repeated the following day. Sessions lasted for 30 min in our initial experiment and 60 min in subsequent experiments, always under low light. The flavor of pellet that was paired with a novel conspecific was the mouse’s less preferred pellet from the training phase, as determined by counting all pellets earned during the last seven days of instrumental training and selecting the pellet that was earned less. The purpose was to bias against any individual pellet preferences. Whenever SIFC was repeated, new novel conspecifics and objects were used.

To determine whether social experience motivated later instrumental responding, mice were returned to the instrumental conditioning chambers for a 15 min probe test conducted in extinction. A socially-motivated mouse would preferentially engage the action predictive of a reward associated with social experience, while a failure to differentiate between actions reflects a failure of social experience to incentivize responding. Response rates on the two apertures were compared. Additionally, preference scores were calculated. Pre-test preference scores were calculated by dividing the responses for the less preferred pellet (to be social pellet) by the responses for the more preferred pellet (to be nonsocial pellet) averaged over the last seven days of training. Any mouse that exhibited a pre-test preference score of <0.5 (an extreme preference for one of the pellets) was excluded from the analysis. Post-test preference scores were calculated by dividing the responses for the social pellet over the nonsocial pellet during the probe test. For the BLA c-Fos investigations, mice were subdivided into responders and nonresponders based on a post-test preference score of > or ≤1.0, respectively.

### Variants of the social conditioning phase

The social pairing conditions were varied to investigate what types of social interactions lead to SIFC. First, one of the pellets was paired with a novel same-strain, same-age female that had been subject to foot shock immediately prior to the social conditioning session using a procedure that increases corticosterone in an observer^13^. The foot shock protocol consisted of a 0.5 mA, two s foot shock delivered every 30 s for a period of five min in a tubular foot shock chamber (5 × 1.5″; SD Labs). In another condition, a pellet was paired with an unfamiliar retired breeding male.

To test the ability of olfactory cues to drive SIFC, one of the pellets was paired with a cotton swab that had been swabbed all over the body, mouth, and anogenital region of a novel female conspecific to trigger affective state discrimination in mice^9^. The other pellet was paired with a clean cotton swab. To recapitulate the warmth that would be generated through huddling in another experiment, one of the pellets was paired with a small heating pad, typically used to aid in recovery from surgery, while the other was paired with a 15 mL falcon tube.

To determine the role of novelty in driving SIFC, one of the pellets was paired with a female conspecific that had been paired with the experimental mouse for 60 min each day for three days prior to the social conditioning session. Thus, the social conspecific in this condition was not novel.

Finally, to determine whether mice must consume food for SIFC to occur, pellets were placed in a covered, plastic dish. The cover had been drilled with multiple holes to allow the mice to smell but not consume the food.

### Behavioral ethograms

Videos of the first 30 min of the social conditioning sessions were scored for relevant behaviors by a single blinded observer. These behaviors are listed in Table 1 and were defined based on prior work characterizing social and nonsocial rodent behaviors^45–47^. Social proximity was defined as the time during which the observer mouse was within a 1″ radius of the demonstrator mouse. Autogrooming and eating were combined as one behavioral measure due to close likeness during observation. Other details are listed in Table 1.

### Three-chamber social interaction and social memory test

Mice were given 10 min to habituate to the middle section of a large chamber (40 × 20 × 25 cm; MazeEngineers) divided into three sections. Dividers between each section were sliding doors to allow for free movement of the animals once opened. After the habituation period, the social interaction test commences. Sliding doors are removed to reveal two lateral chambers containing an acrylic cage (7 cm diameter, 15 cm height) holding either a novel conspecific or no stimulus. Once the mouse leaves the middle section for the first time, 10 min are recorded and scored by a blinded rater for the time in each chamber. A typical mouse will spend more time in the chamber with a novel conspecific than in an empty cage, indicating intact sociality. Following the social interaction test, the experimental mouse is returned to the middle section, and the doors are closed.

For the social memory test, an unfamiliar novel conspecific is placed in the previously empty cage, and doors are removed for another 10 min. Ten min of interaction time are recorded and scored by a blinded rater for time spent in each chamber. A typical mouse will spend more time in the chamber with the unfamiliar conspecific than the familiar conspecific, indicating intact social memory. All videos were analyzed using Jwatcher software v.1.0 by a single rater.

### Viral vector visualization

Mice were transcardially perfused under deep anesthesia with ketamine/xylazine (120/10 mg/kg, i.p., 1 ml/100 g) prior to brain extraction and incubation in 4% paraformaldehyde. Brains were next transferred to 30% w/v sucrose solution, then sectioned on a microtome (Thermo Scientific) held at −15 °C into 50 µm sections, mounted, and coverslipped. mCherry or GFP was visualized using a fluorescence microscope. Immunohistochemistry for mCherry (mouse, 1:1000, Takara; goat-anti-mouse-alexa594, 1:500, Invitrogen) was used to delineate viral vector spread, as needed. Any mice with missed viral vector placement or unilateral infusions were excluded. Representative histological images are provided in the main text, and overlaid images of all mice in a given condition are provided in the supplementary materials. In this case, traces outline all detectable fluorescence at the indicated distance from Bregma in each mouse.

### C-Fos Immunohistochemistry and quantification

Brains were collected as described immediately above 30 min following the probe test. Sections were prepared also as described immediately above and blocked in a 1x PBS solution containing 0.3% Triton X-100 (Sigma), 2% normal goat serum (NGS), and 1% bovine serum albumin (BSA) for 90 min at room temperature prior to incubation in anti-c-Fos primary antibody (SySy; Rb; 1:1000) in a 1x PBS solution containing 2% NGS and 0.3% Triton X-100 at room temperature for 2 hr. Sections then incubated in Alexa Fluor 594 secondary antibody (Jackson Labs; goat-anti-Rb; 1:500) in a 1x PBS solution containing 2% NGS and 0.3% Triton X-100. Finally, sections were mounted and coverslipped.

Sections were imaged on a Nikon 4550 s SMZ18 microscope using NIS-Elements Basic Research imaging software. Fluorescence was quantified by counting c-Fos+ cells within a defined ROI held constant across all images using NIH ImageJ. Between 1–3 sections/mouse were imaged and quantified, and each mouse contributed a single value (the mean of its values) to avoid over-representation of any given animal. Multiple independent cohorts of mice were tested, and values were normalized to the mean of the control group in each cohort (termed the non-responder group) to account for differences in baseline fluorescence between cohorts.

Additional details pertaining to c-Fos quantification for the supplemental figures are provided in the supplementary text.

### Clozapine-N-Oxide (CNO) administration and experimental design

In the region- and projection-specific inactivation experiments, all mice received CNO (Sigma; 1 mg/kg, i.p., in 2% DMSO and saline, 1 ml/100 g), regardless of viral vector condition, to equally expose animals to any unintended consequences of CNO^48^. CNO was always administered 30 min prior to the probe test.

### Statistics and reproducibility

All statistics were performed using Graphpad Prism v.9.0 and SPSS v.27. Repeated measures ANOVA or paired *t*-tests were used to compare response rates or preference scores (before vs. after social conditioning) in behavioral experiments. In the absence of repeating measures, ANOVA or unpaired *t*-tests were applied. In the case of unequal variances, Welch’s ANOVA was used. Tukey’s post hoc tests (or paired *t*-tests in the context of repeated measures) were used in the case of significant interactions or main effects with >2 groups and are indicated in the figures. All comparisons were two-tailed. Corrections for multiple comparisons were not applied throughout.

Pie charts represent the percentage of mice in a given group that display a response preference, defined as ≥1 response for the food pellet associated with a novel conspecific *vs*. the pellet associated with a novel object. Simple linear regressions were used to determine correlations between behaviors during the social conditioning sessions or c-Fos puncta and response preference generated in the SIFC task.

Throughout, a small number of values were >2 standard deviations above the mean and considered outliers and excluded^49^. *p* ≤ 0.05 was considered significant. All experiments were conducted at least twice in independent cohorts of mice, with concordant results. Samples sizes were based on prior experiments and power analyses.

### Reporting summary

Further information on research design is available in the Nature Research Reporting Summary linked to this article.

## Supplementary information

Supplementary Information

Reporting Summary

## Data Availability

Source data are provided with the paper. Databases cited in this study include the Mouse Brain Library (www.mbl.org) and the Allen Brain Atlas (https://mouse.brain-map.org/). Any other materials will be provided upon reasonable request. Source data are provided with this paper.

## Source data

Source Data File
