# Supplementary material for: Social incentivization of instrumental choice in mice requires amygdala-prelimbic cortex-nucleus accumbens connectivity
Source: Nat Commun. 2022 Aug 15;13:4768. doi: 10.1038/s41467-022-32388-9 (PMC9378688; doi:10.1038/s41467-022-32388-9)
Supplement: Supplementary file 1 — Supplementary Information [file 41467_2022_32388_MOESM1_ESM.pdf]

Supplemental Information for:

Social incentivization of instrumental choice in mice requires amygdala-prelimbic cortex-nucleus accumbens connectivity

Henry W. Kietzman, Gracy Trinoskey-Rice, Sarah A. Blumenthal, Jidong D. Guo, Shannon L. Gourley

Contact:
Shannon L. Gourley
Email: shannon.l.gourley@emory.edu

This PDF file includes:

Figures S1 to S5
Supplementary methods
Supplementary references

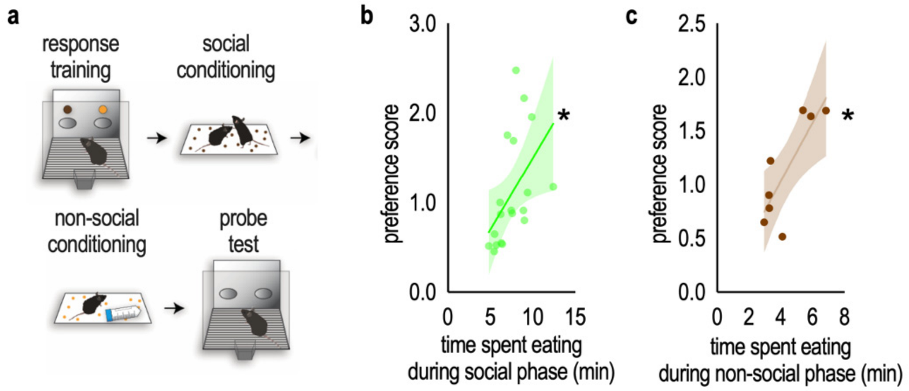

Figure S1. Correlation between consummatory behavior during conditioning sessions with SIFC. a. Schematic of the SIFC task. Mice were trained to nose poke for two food reinforcers. Next, mice were placed in a chamber with either a novel conspecific or object, and with one of the two reinforcers. The next day, each mouse received the other stimulus and pellet. Finally, mice were returned to the operant conditioning chambers used in training, and responding for the reinforcers was quantified. **b**. The amount of time spent eating during the social conditioning phase predicted later preference for the pellet [n=18; simple linear regression: r^2=0.2454, p=0.0310]. **c**. The amount of time spent eating during the non-social conditioning phase similarly predicted later preference for the "social pellet" [n=8; simple linear regression: r^2=0.6679, p=0.0132], presumably because greater consumption of the different pellets allows the mice to better associate the specific sensory properties of the foods with the social conspecific *vs.* object. Panel **b** reproduced from main text Figure 1. Lines represent simple linear regressions, symbols represent individual mice. Shaded area = 90% confidence interval. *p<0.05.

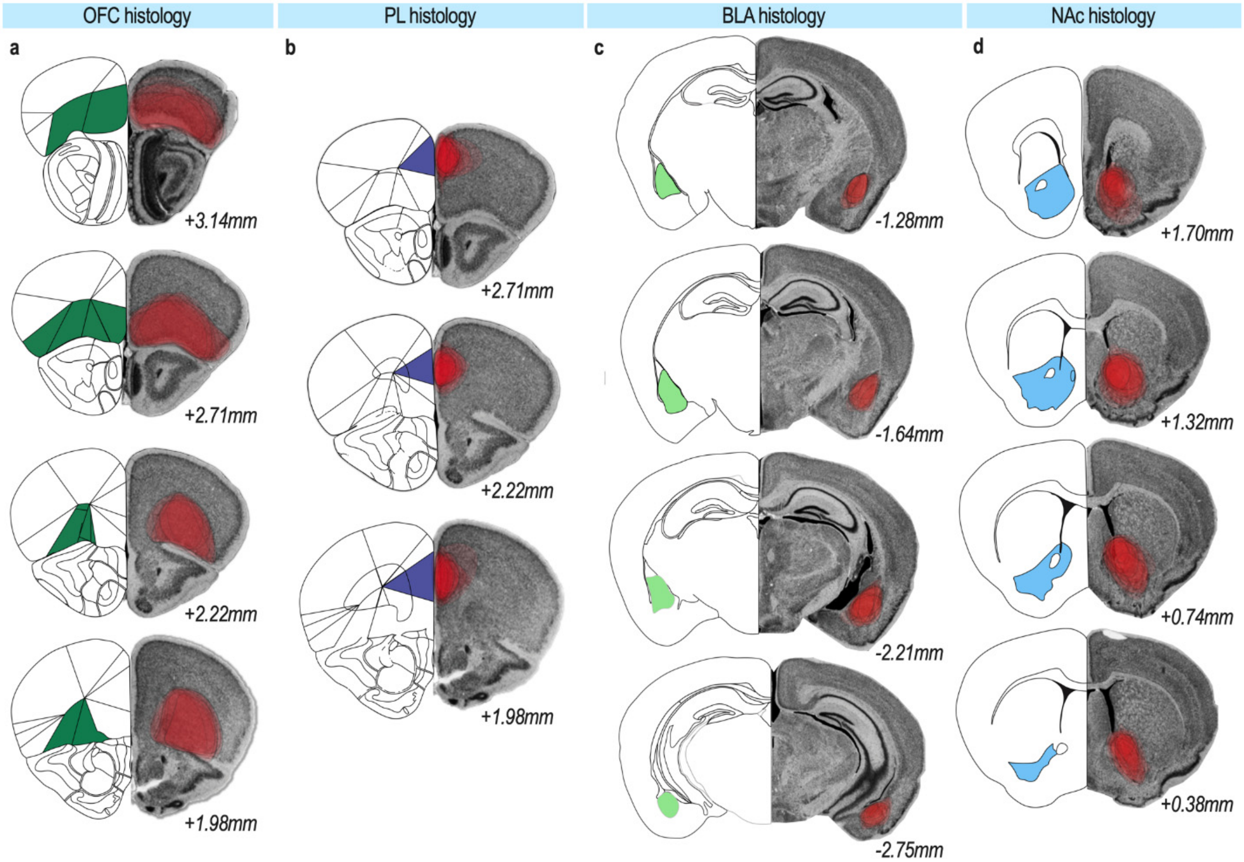

Figure S2. Histology for OFC, PL, BLA, and NAc viral vector infusions. AAVs were infused bilaterally into (**a**) OFC, (**b**) PL, (**c**) BLA, or (**d**) NAc. (**left**) Images from Allen Brain Atlas[1] depicting the OFC (dark green), PL (purple), BLA (light green), or NAc (blue). Distance from bregma is indicated in the figure. (**right**) Overlays of viral vector spread (red) encompassing the OFC, PL, BLA, and NAc from individual mice on images from the Mouse Brain Library[2]. Every mouse in a given condition is represented; accordingly, brighter shades of red signify the localization of the overwhelming number of infusions. All cohorts exhibited consistent viral vector localization; any misses were excluded from analysis.

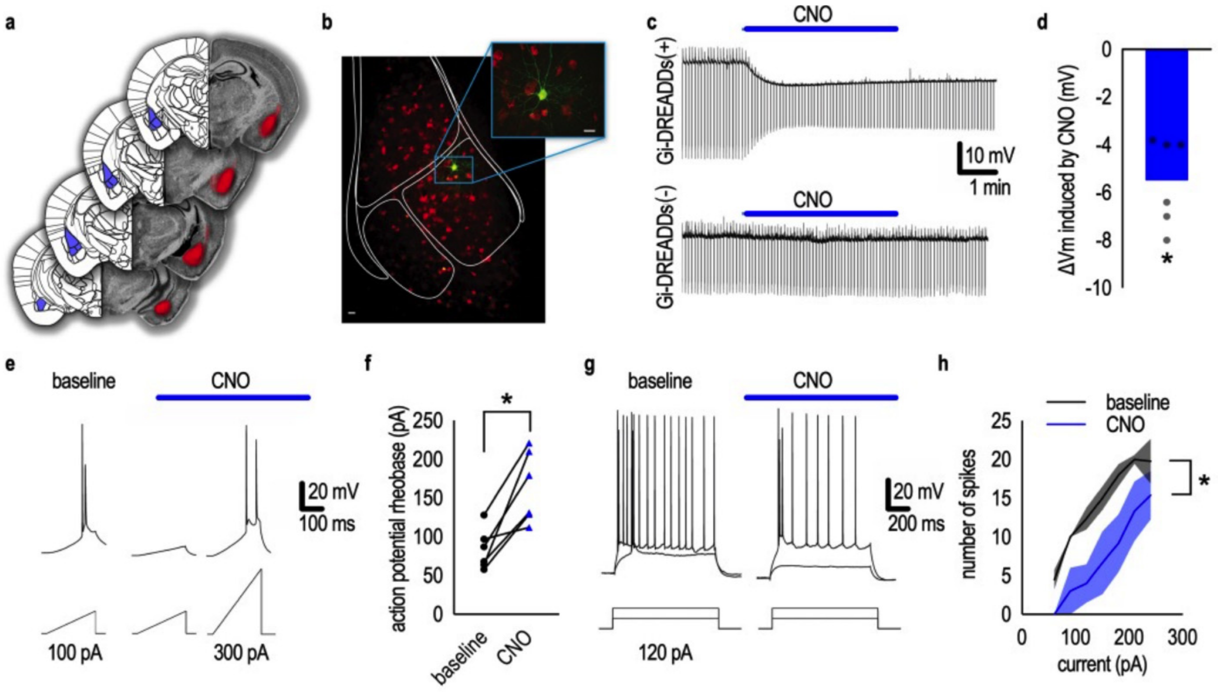

Figure S3. Chemogenetic silencing of PL-projecting BLA neurons. a. To inhibit the activity of projections from the BLA to the PL, mice were infused with viral vectors expressing a Cre-dependent Gi-DREADD into the BLA and a retrograde Cre into the PL. (**left**) Images from Allen Brain Atlas[1] (from left: -1.28 mm, -1.64 mm, -2.12 mm, and -2.72 mm relative to bregma) depicting the BLA (purple). (**right**) Overlays of viral vector spread (red) encompassing the BLA on images from Mouse Brain Library[2]. **b.** Representative image of biocytin-filled (green) BLA neuron expressing Gi-DREADD-mCherry (red); scale bar=20 μm. **c.** In a Gi-DREADD(+) neuron, bath application of CNO (10 μM) induced fast membrane hyperpolarization and decreased input resistance (upper trace). In a Gi-DREADD(-) neuron, CNO did not induce changes of membrane potential and input resistance (lower trace). **d.** CNO-induced hyperpolarization in Gi-DREADD(+) neurons [1-sample t-test; $t_{(5)}$=7.418, p=0.0007]. **e.** Rheobase, the minimal current required to generate action potential, was elevated upon CNO application. **f.** CNO increased rheobase in Gi-DREADD(+) neurons [paired t-test; $t_{(5)}$=4.554, p=0.006]. **g.** CNO decreased the number of action potentials induced by 2 current steps. **h.** CNO decreased action potentials at different current steps [2-way ANOVA with multiple repeating measures: main effect of CNO ($F_{(1,4)}$=21.282, p=0.01), main effect of current ($F_{(3,12)}$=19.026, p<0.001), no current*CNO interaction ($F_{(3,12)}$=2.433, p=0.115)]. Bars represent means, error bars represent SEMs, symbols represent individual neurons, shaded area in (h) represents SEM. *p<0.05. n=6 biologically independent cells from n=4 mice demonstrating similar histology to (**b**), except in (**h**): 5 cells.

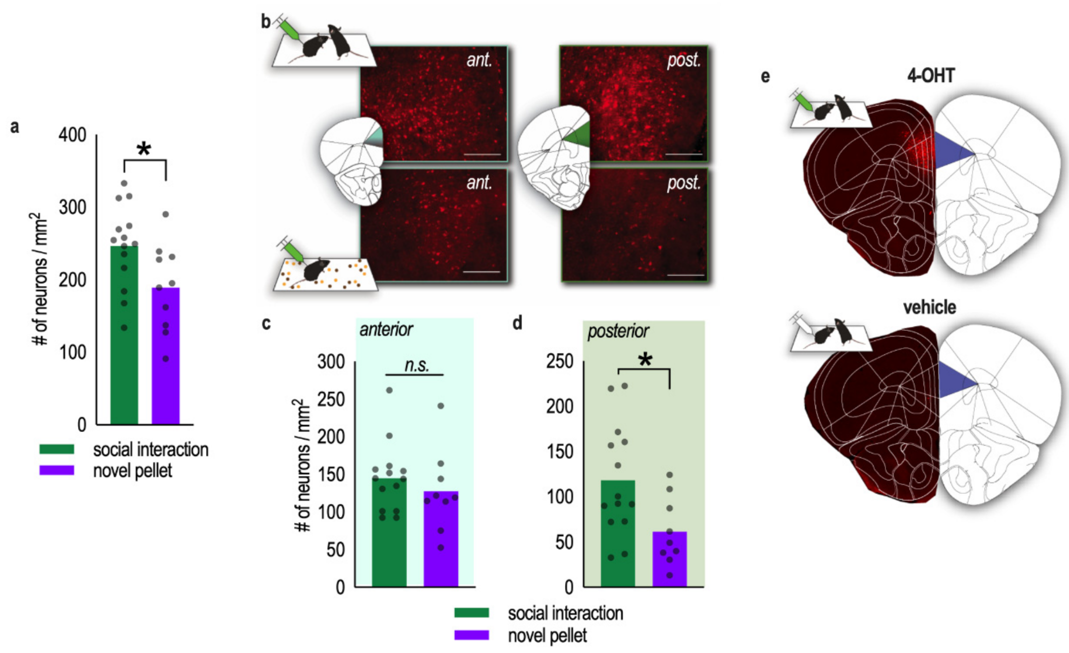

Figure S4. Social interaction triggers immediate-early gene expression in the posterior PL. **a**. We quantified c-Fos+ neurons in the PL following interaction with a novel conspecific *vs.* novel food pellet, revealing more c-Fos+ neurons following interaction with a novel conspecific [n_{soc}=14, n_{pell}=10; unpaired t-test: $t_{(22)}$=2.378, p=0.0265]. **b**. Next, we quantified c-Fos+ neurons in the anterior (~+2.71-1.98mm from bregma) *vs.* posterior (~+1.98-1.32mm from bregma) PL (overlaid coronal sections from Allen Brain Atlas[1]), revealing (**c**) no differences in the anterior PL [n_{soc}=14, n_{pell}=9; unpaired t-test: $t_{(21)}$=0.8336, p=0.4139]. **d**. However, interaction with a novel conspecific triggered greater c-Fos expression in the posterior PL than interaction with a novel food [n_{soc}=14, n_{pell}=9; unpaired t-test: $t_{(21)}$=2.497, p=0.0209]. **e**. These experiments were conducted using 4-OHT-driven Cre, which induced Cre-dependent mCherry (**top**). As a control, we confirmed that mCherry was not expressed in the absence of Cre (signified by vehicle injection at **bottom**). Bars represent means, symbols represent individual mice, *p<0.05. ant.=anterior, post.=posterior. Scale bar=0.5mm.

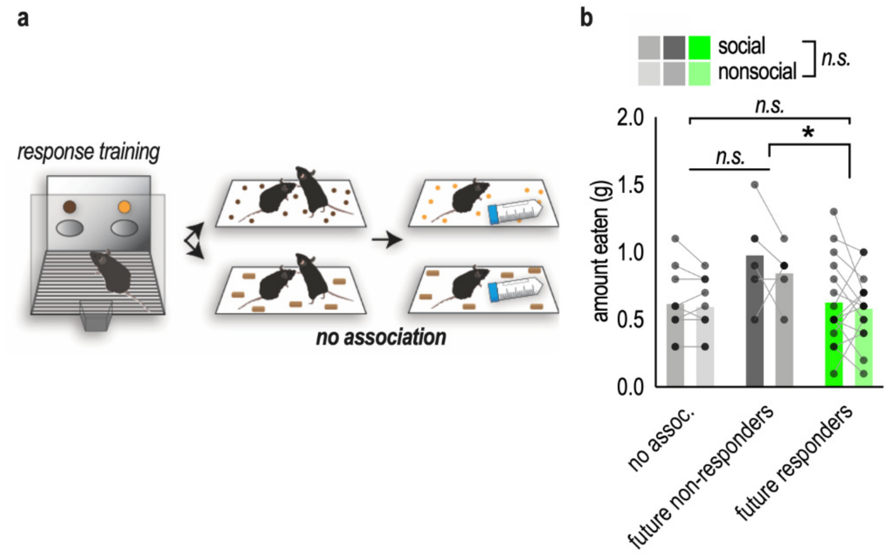

Figure S5. Future "non-responders" eat more than "responders" in the social conditioning phase. a. Schematic of the SIFC task, including "no association" variant. See main text Figure 6 for further explanation. **b**. In the social conditioning phase, future non-responders ate more than the future responders, but no other effects on consumption were detected [$n_{\text{no assoc.}}$=9, $n_{\text{non-resp.}}$=6, $n_{\text{resp.}}$=15; 2-way ANOVA main effect of group: ($F_{(2,27)}$=3.687, p=0.0384), no main effect of pellet ($F_{(1,27)}$=1.897, p=0.182), no pellet*group interaction (F<1)]. Bars represent means, symbols represent individual mice. *p<0.05. No assoc.=no association, non-resp.=non-responder, resp.=responder.

Supplementary Methods

Electrophysiology. Wild type mice were euthanized by rapid decapitation after brief anesthesia with isoflurane. Brains were extracted and sectioned using a vibrating microtome (Leica) into 300 µm sections. Sections containing the BLA were incubated in 95%O_2/5%CO_2 oxygenated 32°C sectioning ACSF for 1 hr before recording (for details, see[3]).

Brain sections were transferred to a recording chamber mounted on the stage of a Leica microscope and perfused with recording ACSF at a rate of ~2 ml/min. BLA→PL projection neurons were identified by the expression of mCherry using a Leica STP6000 epifluorescence microscope. Whole cell patch clamp recordings were performed using a MultiClamp 700B amplifier, a Digidata 1550 digitizer and pClamp 10.6 software (Molecular Devices). Recording pipettes have resistance of 4~6 MΩ when filled with potassium gluconate-based patch solution (in mM): K-Gluconate (130), KCl (2), HEPES (10), $MgCl_2$ (3), phosphocreatine (5), K-ATP (2), and NaGTP (0.2). The patch solution was adjusted to pH 7.3 and had an osmolarity of 280-290 mOsm. 0.3% biocytin was included in the patch solution to stain and localize patched BLA neurons. Membrane potential was current clamped at -60 mV for baseline recordings and input resistance was monitored by observing membrane responses to hyperpolarization current injections (100-200 pA, 0.5 s).

To examine the inhibitory effects of Gi-DREADD activation on BLA neurons, we recorded 3 measures before and during CNO (10 µM) application: **1)** membrane potential hyperpolarization; **2)** rheobase, the minimum current required to generate a single action potential in a ramp current injection protocol (250 ms); and **3)** the number of spikes in response to current injection steps (1 s duration, delta 30-60 pA). Whole-cell access resistances were deemed acceptable if current clamp measurements were <20 MΩ and changed <15% throughout recording. Analysis was performed offline using Clampfit 10.6 (Molecular Devices).

Subjects for Figure S4

Female $Fos^{2A\text{-}iCreER}$ ("TRAP2"[4]) transgenic mice were used, 2-6 months of age. Stimulus mice (that is, mice used as novel conspecifics) were unfamiliar same-strain female mice within one month of age. All mice were bred in-house from Jackson Labs stock, maintained on a 12 hr light cycle (0700 on), and provided food and water *ad libitum* unless otherwise noted.

Surgery and activity-dependent genetic labeling

Transgenic TRAP2 mice were infused into the PL with viral vectors expressing Cre-dependent fluorophores, as in the main text and described in Table 2. Following recovery, they were singly housed in their home cages for one week without disturbance except for standard laboratory care. Next, mice were habituated to a testing room for 30 mins, and finally, placed for five mins in a clean cage with either a novel, same-age, same-sex conspecific or a novel food pellet. Mice remained in the quiet, dim testing room for 90 mins, at which point they were injected with 4-OHT, as described previously[5] (Sigma; 50 mg/kg, *i.p.*, in 5% DMSO and saline/2% Tween-80, 1 ml/100 g). They then remained without disturbance for at least four more hours, in an effort to minimize immediate-early gene expression unrelated to the novel conspecific or object. Mice were then returned to the animal vivarium until imaging experiments roughly 2 months later. Additional mice were treated with either 4-OHT as above or vehicle to confirm the expected absence of mCherry upon vehicle administration (Figure S4e).

Histological quantification of c-Fos

Mice were euthanized and brain sections were prepared as in main text. mCherry+ cells were quantified, representing c-Fos+ cells. Sections were imaged using a Nikon 4550s SMZ18 fluorescent microscope held at consistent magnification, and each image was processed using NIS-Elements Basic Research imaging software. Two sections/mouse were collected from both the anterior PL (~+2.71-1.98mm from bregma) and posterior PL (~+1.98-1.32mm from bregma),

and an average c-Fos density/compartment/mouse was calculated. Boundaries of the PL were determined using the Allen Brain Atlas[1]. Fluorescence was quantified by a single rater blinded to group by counting mCherry+ cells within ROIs held constant across all images using NIH ImageJ.

Supplemental References

1. Lein ES, Hawrylycz MJ, Ao N, Ayres M, Bensinger A, Bernard A, Boe AF, Boguski MS, Brockway KS, Byrnes EJ, Chen L, Chen L, Chen T-M, Chi Chin M, Chong J, Crook BE, Czaplinska A, Dang CN, Datta S, Dee NR, Desaki AL, Desta T, Diep E, Dolbeare TA, Donelan MJ, Dong H-W, Dougherty JG, Duncan BJ, Ebbert AJ, Eichele G, Estin LK, Faber C, Facer BA, Fields R, Fischer SR, Fliss TP, Frensley C, Gates SN, Glattfelder KJ, Halverson KR, Hart MR, Hohmann JG, Howell MP, Jeung DP, Johnson RA, Karr PT, Kawal R, Kidney JM, Knapik RH, Kuan CL, Lake JH, Laramee AR, Larsen KD, Lau C, Lemon TA, Liang AJ, Liu Y, Luong LT, Michaels J, Morgan JJ, Morgan RJ, Mortrud MT, Mosqueda NF, Ng LL, Ng R, Orta GJ, Overly CC, Pak TH, Parry SE, Pathak SD, Pearson OC, Puchalski RB, Riley ZL, Rockett HR, Rowland SA, Royall JJ, Ruiz MJ, Sarno NR, Schaffnit K, Shapovalova NV, Sivisay T, Slaughterbeck CR, Smith SC, Smith KA, Smith BI, Sodt AJ, Stewart NN, Stumpf K-R, Sunkin SM, Sutram M, Tam A, Teemer CD, Thaller C, Thompson CL, Varnam LR, Visel A, Whitlock RM, Wohnoutka PE, Wolkey CK, Wong VY, Wood M, Yaylaoglu MB, Young RC, Youngstrom BL, Feng Yuan X, Zhang B, Zwingman TA, Jones AR. Genome-wide atlas of gene expression in the adult mouse brain. Nature. 2007;445(7124):168-76.
2. Rosen GD, Williams, A.G., Capra, J.A., Connolly, M.T., Cruz, B., Lu, L., Airey, D.C., Kulkarn,i K., Williams, R.W. . The Mouse Brain Library @ www.mbl.org. Int Mouse Genome Conference. 2000;14(166).
3. Guo J-D, O'Flaherty BM, Rainnie DG. Serotonin gating of cortical and thalamic glutamate inputs onto principal neurons of the basolateral amygdala. Neuropharmacology. 2017;126:224-32.
4. DeNardo LA, Liu CD, Allen WE, Adams EL, Friedmann D, Fu L, Guenthner CJ, Tessier-Lavigne M, Luo L. Temporal evolution of cortical ensembles promoting remote memory retrieval. Nature Neuroscience. 2019;22(3):460-9.
5. Xing B, Mack NR, Guo K-M, Zhang Y-X, Ramirez B, Yang S-S, Lin L, Wang DV, Li Y-C, Gao W-J. A Subpopulation of Prefrontal Cortical Neurons Is Required for Social Memory. Biological Psychiatry. 2020.
