# Supplementary material for: Social incentivization of instrumental choice in mice requires amygdala-prelimbic cortex-nucleus accumbens connectivity
Source: Nat Commun. 2022 Aug 15;13:4768. doi: 10.1038/s41467-022-32388-9 (PMC9378688; doi:10.1038/s41467-022-32388-9)
Supplement: Supplementary file 2 — Reporting Summary [file 41467_2022_32388_MOESM2_ESM.pdf]

nature portfolio

Corresponding author(s): Shannon Gourley

Last updated by author(s): Jul 14, 2022

Reporting Summary

Nature Portfolio wishes to improve the reproducibility of the work that we publish. This form provides structure for consistency and transparency in reporting. For further information on Nature Portfolio policies, see our Editorial Policies and the Editorial Policy Checklist.

Statistics

For all statistical analyses, confirm that the following items are present in the figure legend, table legend, main text, or Methods section.

n/a	Confirmed	
☐	☒	The exact sample size (*n*) for each experimental group/condition, given as a discrete number and unit of measurement
☐	☒	A statement on whether measurements were taken from distinct samples or whether the same sample was measured repeatedly
☐	☒	The statistical test(s) used AND whether they are one- or two-sided<br>*Only common tests should be described solely by name; describe more complex techniques in the Methods section.*
☐	☒	A description of all covariates tested
☐	☒	A description of any assumptions or corrections, such as tests of normality and adjustment for multiple comparisons
☐	☒	A full description of the statistical parameters including central tendency (e.g. means) or other basic estimates (e.g. regression coefficient) AND variation (e.g. standard deviation) or associated estimates of uncertainty (e.g. confidence intervals)
☐	☒	For null hypothesis testing, the test statistic (e.g. *F*, *t*, *r*) with confidence intervals, effect sizes, degrees of freedom and *P* value noted<br>*Give P values as exact values whenever suitable.*
☒	☐	For Bayesian analysis, information on the choice of priors and Markov chain Monte Carlo settings
☒	☐	For hierarchical and complex designs, identification of the appropriate level for tests and full reporting of outcomes
☒	☐	Estimates of effect sizes (e.g. Cohen's *d*, Pearson's *r*), indicating how they were calculated

Our web collection on statistics for biologists contains articles on many of the points above.

Software and code

Policy information about availability of computer code

Data collection	We used JWatcher version 1.0 for video analyses
Data analysis	All statistics were performed using Graphpad Prism v.9.0 and SPSS v.27.

For manuscripts utilizing custom algorithms or software that are central to the research but not yet described in published literature, software must be made available to editors and reviewers. We strongly encourage code deposition in a community repository (e.g. GitHub). See the Nature Portfolio guidelines for submitting code & software for further information.

Data

Policy information about availability of data

All manuscripts must include a data availability statement. This statement should provide the following information, where applicable:

- Accession codes, unique identifiers, or web links for publicly available datasets
- A description of any restrictions on data availability
- For clinical datasets or third party data, please ensure that the statement adheres to our policy

Source data are provided with the paper. Databases cited in this study include the Mouse Brain Library (www.mbl.org) and the Allen Brain Atlas (https://mouse.brain-map.org/).

Field-specific reporting

Please select the one below that is the best fit for your research. If you are not sure, read the appropriate sections before making your selection.

☒ Life sciences ☐ Behavioural & social sciences ☐ Ecological, evolutionary & environmental sciences

For a reference copy of the document with all sections, see nature.com/documents/nr-reporting-summary-flat.pdf

Life sciences study design

All studies must disclose on these points even when the disclosure is negative.

Sample size	Samples sizes were based on prior experiments and power analyses.
Data exclusions	Throughout, a small number of values were >2 standard deviations above the mean and considered outliers and excluded
Replication	Experiments were conducted at least twice, with concordant results.
Randomization	mice were randomized to group
Blinding	Blinding occurred during collection, but not analyses because it would occlude our ability to detect outliers.

Reporting for specific materials, systems and methods

We require information from authors about some types of materials, experimental systems and methods used in many studies. Here, indicate whether each material, system or method listed is relevant to your study. If you are not sure if a list item applies to your research, read the appropriate section before selecting a response.

Materials & experimental systems

n/a	Involved in the study	
☐	☒	Antibodies
☒	☐	Eukaryotic cell lines
☒	☐	Palaeontology and archaeology
☐	☒	Animals and other organisms
☒	☐	Human research participants
☒	☐	Clinical data
☒	☐	Dual use research of concern

Methods

n/a	Involved in the study	
☒	☐	ChIP-seq
☒	☐	Flow cytometry
☒	☐	MRI-based neuroimaging

Antibodies

Antibodies used

Primary antibodies used:

Mcherry, mouse, Takara Cat. #632543, Lot# 1802187A
c-fos, Rabbit, SySy Cat. #226 008, Clone# Rb108B5, Lot#7-83

Secondary antibodies used:

goat anti-mouse-Alexa Fluor 594, Invitrogen, Cat.# A32742, Lot# 1832034
goat anti-rabbit-Alexa Fluor 594; Jackson Immunoresearch, Cat.# 111-585-144, Lot# 118895

Validation

Validations per source websites:

Mcherry, mouse, Takara Cat. #632543, Lot# 1802187A

Validation: Tested by western blot analysis. Lysates (10 µl; equivalent to 35,000 cells) from untransfected HEK 293 cells and from HEK 293 cells transiently transfected with a CMV-driven expression vector encoding mCherry was resolved on a 12% SDS polyacrylamide gel and then transferred to a membrane certified for western blot applications

c-fos, Rabbit, SySy Cat. #226 008, Clone# Rb108B5, Lot#7-83

Validation: 12-O-tetradecanoylphorbol-13-acetate (TPA) treated HeLa cell lysate (1:5000)
Indirect immunostaining of PFA fixed mouse hippocampus section with rabbit anti-cFos (dilution 1:5000)

goat anti-mouse-Alexa Fluor 594, Invitrogen, Cat.# A32742, Lot# 1832034

Validation: Immunofluorescent analysis of tubulin in U2OS cells. The cells were fixed with 4% formaldehyde for 20 mins, permeabilized with 0.5% Triton X-100 in PBS for 20 mins, washed 3X in PBS and blocked with 3% BSA in PBS for 30 mins at RT. Cells were stained with a tubulin antibody at a dilution of 1:1000 in 3% BSA in PBS for 1 hr at RT, washed 3X in PBS and then incubated with Invitrogen Alexa Fluor Plus 594 goat anti-rabbit IgG secondary antibody

goat anti-rabbit-Alexa Fluor 594; Jackson Immunoresearch, Cat.# 111-585-144, Lot# 118895

Validation: Based on immunoelectrophoresis and/or ELISA, the antibody reacts with whole molecule rabbit IgG. It also reacts with the light chains of other rabbit immunoglobulins. No antibody was detected against non-immunoglobulin serum proteins. The antibody has been tested by ELISA and/or solid-phase adsorbed to ensure minimal cross-reaction with human, mouse, and rat serum proteins

Animals and other organisms

Policy information about studies involving animals; ARRIVE guidelines recommended for reporting animal research

Laboratory animals	Experimental mice were female C57BL/6 mice 2-6 months of age. Stimulus mice (that is, mice used as novel conspecifics) were unfamiliar same-strain females within 1 month of age. The one exception is an experiment in which we used mature (6-12 months) unfamiliar breeding males from our colony, also bred on a C57BL/6 backrgound. For some Fos investigations, female Fos2A-iCreER ("TRAP2") mice, 2-6 months of age, were used for experimental and stimulus mice.
Wild animals	No wild animals were used.
Field-collected samples	No field samples were used.
Ethics oversight	Procedures were approved by the Emory University IACUC.

Note that full information on the approval of the study protocol must also be provided in the manuscript.
